# INTEREST GROUP SESSION—COMMUNITY COLLEGE: THE ROLE OF COMMUNITY COLLEGES IN WORKFORCE DEVELOPMENT AND TRANSFER WITHIN GERONTOLOGY

**DOI:** 10.1093/geroni/igz038.898

**Published:** 2019-11-08

**Authors:** Jan Abushakrah, Michael A Faber

**Affiliations:** Portland Community College, Portland, Oregon, United States

## Abstract

This symposium will highlight how innovative and often non-traditional Community College Gerontology students are motivated to seek career shifts and intentional training and comprehensive education. Understanding and applying this approach allows students to build on their prior skills, especially caregiving experience, to advance to more professional roles in the field of Gerontology. During this symposium we will focus on the Applied Gerontology aspect of Community College programs, including short-term certificates and other approaches related to and informed by the rapidly evolving workforce development in the field of aging. Selected Community College and University representatives, who understand and are leaders in the field of Applied Gerontology, will highlight models of existing Community College and University partnerships/collaboration that work, as well as provide models of other applied approaches. In addition, there will be an opportunity for robust dialog between Community Colleges and four-year Colleges and Universities – both on developing other effective Applied Gerontology approaches, and on creating even better partnerships and collaboration. This symposium will appeal to professionals working in both two and four-year systems of higher education. Colleges and Universities desiring to develop or enhance relationships with area Community Colleges will find this session especially helpful.

